# The Use of Self-Study and Matrix Training to Teach Sign Language

**DOI:** 10.1007/s40616-026-00228-2

**Published:** 2026-04-27

**Authors:** Sarah E. Vesely, Sarah E. Frampton

**Affiliations:** Psychology Department, University of Nebraska Omaha, 6001 Dodge St, Omaha, NE USA

**Keywords:** Augmentative and alternative communication, Matrix, Sign language, Stimulus pairing observation procedure, Verbal behavior

## Abstract

Augmentative and alternative communication (AAC) systems allow individuals with vocal speech delays to meet their wants and needs. However, when signs are used as AAC, the implementers and caregivers must also learn signs. Longo et al. *Behavioral Interventions, 37*(3), 713–731 (2022) found that a self-study training (SST) in which adult participants observed videos paired with text was both efficacious and efficient in teaching eight component and 16 compound signs, arranged in a matrix. We systematically replicated SST, with changes to the matrix design and targets evaluated. The dependent variables were text-sign intraverbal and sign-vocal tacts for component and compound targets. Performance in a role play condition was also evaluated before and after training. SST was implemented with three adult participants using a multiple probe design across behaviors (submatrices). For the first two participants, SST of six compound, diagonal targets led to the acquisition of 30 untrained compounds and 12 component targets across modalities (i.e., text-sign intraverbals, sign-vocal tacts, listener responses) and contexts (i.e., role play). Similar results were obtained with the third participant, though acquisition of submatrix 2 was observed following intervention with submatrix 1. These results have implications for designing training to support implementers teaching sign as AAC.

**Supplementary Information:**

The online version contains supplementary material available at 10.1007/s40616-026-00228-2.

It is estimated that 30% of children with a diagnosis of autism spectrum disorder (ASD) do not acquire functional vocal speech (Tager-Flusberg & Kasari, 2013). For these individuals, augmentative and alternative communication (AAC) can create new opportunities for communication through alternative systems (Beukelman & Light, 2020; McNaughton et al., 2008; Sigafoos et al., 2014). AAC consists of aided and unaided techniques, defined by whether external materials are necessary (Iacono et al., 2016; Sigafoos et al., 2014). Sign language is an example of an unaided AAC system, which has been used for decades to support individuals with ASD (Nunes, 2008) as they learn a variety of verbal operants (e.g., Bartman & Freeman, 2003; Falcomata et al., 2013; Scattone & Billhofer, 2008; Valentino & Shillingsburg, 2011).

To teach sign as a form of AAC, caregivers and staff members must also be trained as both speakers and listeners in sign. The implementers must produce the signs in the presence of establishing operations relevant to the client (as in a mand), nonverbal stimuli (as in a tact), and verbal stimuli (as in an intraverbal). These responses by the implementers will also likely be controlled by textual or picture stimuli included within program materials (e.g., data sheets listing targets). Implementers must also serve as competent listeners to quickly provide the putative reinforcer when a sign is emitted to complete the verbal episode (Skinner, 1957).

Unfortunately, parents of children with ASD have reported a lack of trained professionals to support their child learning sign (McNaughton et al., 2008). Efforts required to learn sign have been noted as a concern when considering use of sign as AAC (Bonvillian et al., 2020; Sundberg & Sundberg, 1990). Though curricula exist for instruction in American Sign Language (ASL; Rosen, 2010), these approaches may be too time-intensive and broad for implementers seeking to learn enough signs to support an early communicator (Napier et al., 2007). Curricular instruction in sign would not likely be feasible in clinical settings in which time for training is limited (Love et al., 2009) and turnover is high for direct care staff (Cymbal et al., 2022).

Researchers have investigated group-based approaches to sign instruction (Chadwick & Jolliffe, 2009; Fitzgerald et al., 1984; Parsons et al., 2017) that utilize components of behavior skills training (BST; Sarokoff & Sturmey, 2004). Parsons et al. (2017) trained staff members working with students that used sign as AAC with a package that included a verbal description of how to produce each sign and a group practice session with peer feedback. Following training, participants demonstrated increased accuracy in producing sign under control of dictated words (vocal-sign intraverbals) and rated the training as highly socially valid. As a limitation, Parsons et al. noted the training format was time-intensive due to the repeated practice trials. Overall, this group format may be a viable option for a team of staff members serving clients that sign, but too labor intensive to repeat with each new hire. Training packages that can be completed individually with minimal trainer involvement may permit more flexible deployment.

Longo et al. (2022) compared two such approaches, equivalence-based instruction and self-study, to teach sign using a group design. Participants in both groups were tested on the formation of eight three-member equivalence classes composed of a picture of a noun or verb (A), a video of the term in ASL (B), and the printed word (C). Four of the classes corresponded to nouns (e.g., truck, dollar, ball, egg) and four to verbs (e.g., touch, throw, spin, blow), arranged in a four-by-four matrix. Participants in the equivalence-based instruction group were taught to select videos of signs in the presence of the picture (AB relation) using a matching-to-sample procedure with systematic mastery criteria. Participants in the self-study group were instructed to watch a video of each sign in the presence of the printed word (AC relation) as many times as they liked until they determined they were done (i.e., no systematic mastery criteria; no explicit responses required). Pre- and posttests for all 24 participants evaluated video-picture (BA), video-word (BC), and word-video (CB) relations in a matching-to-sample format. Experimenters also evaluated text-sign intraverbals for the eight component words (i.e., signing when shown text), vocal-sign intraverbals (i.e., signing after dictated words) with the 16 noun-verb compounds, and listener responding (i.e., demonstrating responses following signs) with the same 16 noun-verb compounds.

Participants in both groups demonstrated moderately high levels of correct responding during the matching-to-sample baseline, but near zero levels of correct responding for the speaker and listener responses. Following training, both groups demonstrated criterion-level performance on the matching-to-sample posttest. Participants in the equivalence-based instruction group demonstrated criterion-level performance in the single word text-sign intraverbal posttest on the first attempt; most participants in the self-study group had to repeat the intervention before this level of performance was observed. Participants in both groups demonstrated criterion-level performance with the sign-vocal tact and listener posttest with compound signs, slightly higher levels of performance were observed in the equivalence-based instruction group. Participants in the equivalence-based instruction group rated the training higher than participants in the self-study group on a social validity survey. Though the difference was statistically significant, the self-study group still provided favorable ratings. In terms of efficiency, the participants in the self-study group (*M*= 8.0 minutes) completed training almost three times faster than the participants in the equivalence-based instruction group (*M* = 22.5 minutes). In summary, both approaches produced substantial increases in speaker and listener responses related to sign, in a relatively short period of time.

Self-study procedures seem to be a promising avenue for staff members or caregivers to learn specific signs, as the approach is efficacious, efficient, and feasible. As self-study consisted only of instructing participants to watch videos of signs paired with a word, they could be easily reproduced in low resource settings and customized to clients’ sign repertoires. However, more research is needed to evaluate this approach to sign instruction.

The present study aimed to systematically replicate the self-study training (SST) package used by Longo et al. (2022) with variations to the dependent variables evaluated, expansion of the role play, design of the matrix, and research design. As it would be unlikely that clients learning sign would be vocalizing, we reversed the dictated word to sign relation into a sign-vocal tact. To take a step toward a more applied evaluation, we modified the speaker and listener probes used by Longo et al. to resemble a role play condition. We expanded the matrix to include more components and applied a non-overlap training approach to teach the compound sign targets on the interior diagonal of the matrix (Frampton & Axe, 2023). To evaluate the effects of SST, the matrix was divided into three submatrices and functional control was evaluated in a multiple probe design across submatrices (e.g., Axe & Sainato, 2010).

Thus, the present study asked what are the effects of SST on sign-vocal tact and text-sign intraverbal relations for (1) the diagonal (i.e., trained) and non-diagonal (i.e., untrained) targets in each submatrix, (2) the recombinative generalization targets (i.e., not included in any of the targeted submatrices), and (3) the components (i.e., terms on each axis of the matrix)? Also, what are the effects of SST on text-sign and listener response relations in a role-play context? As additional measures, the duration of SST was collected to determine overall efficiency, and social validity was evaluated via a questionnaire.

## Methods

### Participants

Participants were recruited from among undergraduate Psychology students at a public, metropolitan Midwestern university. Recruitment was completed using an online research study platform that allowed students to choose among available research studies for course credit. All participants had to be 19 years or older, enrolled in undergraduate courses, English-speaking, report no developmental or cognitive delays, and report little to no exposure to American Sign Language. Completion of the study resulted in one credit within the research management system for every 30 minutes spent participating, regardless of performance on tasks. Credits were exchanged into points within courses at the discretion of instructors. All procedures were approved by the university’s institutional review board prior to initiating the study with any participants. One Black, one White, and one Hispanic female between the ages of 19 and 21 participated in the study.

### Setting, Materials, and Stimuli

All sessions were conducted in a private room (5 m by 5 m) that contained three tables and three chairs. Supplemental Materials A–C display illustrations of the Microsoft PowerPoint slides used in all training and testing conditions. PowerPoint slides were presented on either a ThinkPad 2018 or MacBook Air 2022 laptop. PowerPoint slides for the text-sign intraverbal probes included a written instruction to produce the sign for the indicated word. PowerPoint slides for the sign-vocal tact probes included a video of a sign on a loop with the written instruction to label it. A training trial in SST included three different PowerPoint slides; one with a written instruction to produce a compound sign, one with the printed term and the compound sign video on a loop, and then another with the same written instruction and no video. During training and probe tasks, the participants faced the computer and sat to the left of the researcher.

For the role play condition, 12 white notecards (7.62 x12.7 cm) with hand-written compound words on the unlined side were used to recreate the types of teaching materials that implementers may have access to when teaching sign as AAC. For the listener responding role play, materials corresponding to the sign targets were arranged on the table to the participant’s left. The researcher laid out two of each of the materials including: chips ahoy chocolate chip cookie in a zip-lock bag, wrapped chocolate candy, Elmer’s glue stick, Barbie doll, white notebook paper (21 × 29.7 cm), Thomas the Train, dry erase marker, solo cup, and a roll of tape. During the roleplay conditions, the participant faced the researcher and had the table directly to their left side. Throughout all sessions, an iPad was placed diagonally in front of the participant to collect an audio and video recording of the session.

#### Target Selection and Matrix Design

**Fig. 1:**
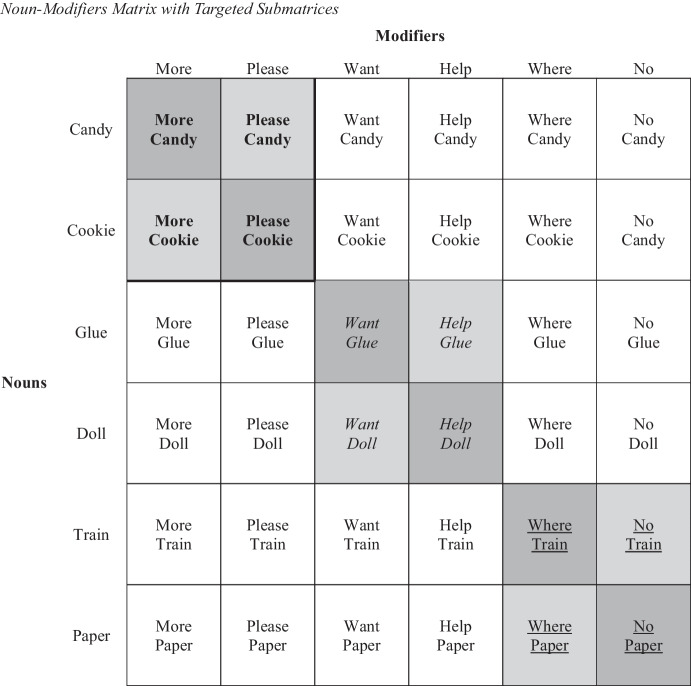
Noun-modifiers matrix with targeted submatrices. *Note.* Diagonal targets are shaded in dark gray, non-diagonal targets in the submatrices are shaded in light gray, and recombinative generalization targets are shaded in white. The four targets in submatrix 1 are bolded, submatrix 2 are italicized, and submatrix 3 are underlined

Figure 1 includes a visual depiction of the matrix and Table 1 includes a description of all the signs included. The first author developed the signs and broke them down into components using her previous coursework in ASL and referencing to a popular online sign language website, Signing Savvy. One axis of the matrix consisted of nouns (cookie, candy, glue, doll, paper, and train) and the other of modifiers (more, please, want, help, no, and where). The 36 combinations of the component signs found in the interior of the matrix were referred to as compounds. The matrix was then divided into three submatrices, each including two nouns and two modifiers, for a total of four compounds. Within those submatrices, the two compounds on the diagonal were selected for training, referred to as diagonal targets, the two non-diagonal targets were only tested. The remaining 24 compound targets, not included in a submatrix, were referred to as the recombinative generalization targets. The 24 compound targets were then divided into two groups based on whether they would be used for the role-play or listener-responding conditions. To do this, the first author identified the modifiers that would be easiest to demonstrate as a listener (i.e., want, more, please, and where) and those that would be more difficult (i.e., no and help). For example, if the researcher signed “where cookie,” a corresponding response of pointing to the item could occur. However, if the researcher signed “no cookie,” there would not be a clear response for the learner to engage in other than not responding. The first author attempted to distribute the corresponding nouns/items evenly across the two groups.

**Table 1:** Component sign characteristics

Sign	Must have characteristics
More	1. Flat O hand shape with both hands 2. Palms face each other 3. Two finger taps 4. Located around chest level
Please	1. 5 hand shape with dominant hand 2. Palm facing chest 3. One full circle 4. Located at center of chest
*Want*	1. Claw hand shape with both hands 2. Palms facing up 3. One pull of hands toward body 4. Located somewhere between chest and waist
*Help*	1. Dominant hand: A handshape; Non-dominant hand: 5 handshape 2. Dominant hand: palm and fingers facing towards body; Non-dominant hand: palm facing up 3. Dominant hand moves to nondominant hand, then lift 4. Located somewhere between chest and waist
Where	1. 1-handshape with dominant hand 2. Palm facing away from body 3. Tilt side to side two times 4. Located in front of body between shoulder and waist
No	1. Closed 3- handshape (pointer and middle finger touching thumb) with dominant hand 2. Palm facing out 3. Fingers and thumb tap two times 4. Located somewhere between shoulder and waist
**Candy**	1. 1- handshape with dominant hand 2. Start with palm facing in 3. Twist wrist three times 4. Located on cheek (same side as dominant hand)
**Cookie**	1. Dominant hand: C- handshape; Nondominant hand: 5 handshape 2. Dominant hand: Palm facing down/ nondominant hand; Non-dominant hand: Palm facing up/ dominant hand 3. Twist dominant hand twice on nondominant hand 4. Located somewhere between chest and waist
*Glue*	1. Dominant hand: A- handshape; Non-dominant hand: 5 handshape 2. Dominant hand: Palm facing out; Non-dominant hand: Palm facing up 3. Brush the thumb of the dominant hand on to nondominant hand away from body 4. Located somewhere between chest and waist
*Doll*	1. X- handshape with dominant hand 2. Palm facing nondominant side 3. X handshape brushes down twice 4. Located on nose
Train	1. U handshape with both hands 2. Both palms facing down 3. Dominant hand moves back and forth on nondominant hand two times 4. Located somewhere between chest and waist
Paper	1. 5 handshape with both hands 2. Palm of dominant hand faces down; Palm of nondominant hand faces up 3. Dominant hand brushes against nondominant hand two times 4. Located somewhere between chest and waist

In alignment with Fig. 1, the component targets are bolded for submatrix 1, italicized for submatrix 2, and underlined for submatrix 3

## Dependent Variables

The primary dependent variable was the percentage of correct compound and component text-sign intraverbal responses. Text-sign intraverbal responses consisted of the production of the sign(s) that corresponded with the text presented on a PowerPoint slide. For example, signing “more cookie” when shown the text *more cookie* or signing “cookie” when shown the text *cookie*. To be scored as correct, responses had to include the four necessary characteristics for each individual sign. Any attempt that did not include all characteristics was scored as incorrect. Table 1includes a description of the required characteristics for each individual sign.

The secondary dependent variable was the percentage of correct compound and component sign-vocal tact responses. Sign-vocal tact responses consisted of a vocal response emitted under control of the sign(s) presented on a video embedded in a PowerPoint slide. For example, saying “more cookie” when shown the compound sign of “more cookie” or “cookie” when shown the single sign of “cookie.”

As an additional measure, the percentage of correct text-sign intraverbal compound responses was evaluated in the context of a role play. The text was presented on an index card to resemble materials used in teaching sessions with children with ASD. The criteria for scoring were the same as described above.

The percentage of correct listener responses was also evaluated in the role play context. To be scored as correct, the response had to be initiated within 10 seconds of the experimenter’s compound sign and had to correspond with the compound sign. For example, if the researcher signed “where train,” any response the participant made to indicate where the train was, was scored as correct. Any response that included manipulations of the object that did not correspond with the signed compound was scored as incorrect. For example, if the participant pointed to the location of the candy following the sign “where train,” the researcher scored it as incorrect.

As an additional measure, the total duration of the SST condition was collected. The researcher started a timer as the first slide of training for each submatrix was presented and stopped the timer once the participant’s responding had met the SST mastery criteria for the submatrix. The researcher combined all the individual self-study duration measures for all three submatrices into a total duration measure.

## Inter-Observer Agreement and Procedural Fidelity

Inter-observer agreement (IOA) data was collected independently by a trained second observer for 33.33–53% of trial blocks for each condition and each participant. Like Longo et al. (2022), observers underwent training and were tested on producing the sign and describing the characteristics before they could collect data. The primary researcher (first author) was already proficient in sign having completed four semesters of an undergraduate ASL course. The observer watched the same 12 component sign videos that were presented to the participants. Once the observer researcher could independently produce each sign, they were instructed to study all the necessary characteristics for each sign. Finally, the primary researcher tested the observer on the production and tacting of component signs. The table with the characteristics of each sign was available to the observer when scoring to promote accuracy.

All sessions were audio and video recorded for data collection purposes. The primary researcher selected sessions for IOA coding using a random number generator. An agreement occurred when both observers coded the response in the same way. A disagreement was scored when the observers coded the response differently. Trial-by-trial IOA was collected by comparing the scoring on each trial. The total number of agreements was divided by the total number of trials and multiplied by 100. During the pre- and post-training probes (i.e., recombinative generalization and roleplay targets), IOA was 100%. During Submatrices 1–3 baseline and post-training probes, the mean IOA across participants was 94.44% (range 83.33–100%). There were two disagreements related to coding compound sign-vocal tact trials for participant 1. Lastly, mean IOA across participants during SST was 97.22% (range 91.67–100%). Observers disagreed on one SST trial for participant 3.

The second observer also collected procedural fidelity (PF) data for the same randomly selected sessions using a checklist. Items on the fidelity checklist reflected important procedures for each condition including presenting instructions, interacting as prescribed in the protocol, and withholding feedback for responses. Supplemental Materials D–F include the PF checklists used in each condition. The observer used a “+” to denote correct adherence, and a “−” when the researcher deviated from the protocol step. To produce a PF score, the number of steps completed correctly were divided by the total number of steps on the PF checklist and multiplied by 100. During the pre- and post-training probes (i.e., recombinative generalization and roleplay targets) and submatrices 1–3 baseline and post-training probes, PF was 100% across participants. During SST, mean PF was 98.61% (range 95.83–100%) across participants. The procedural errors were made during SST with P3 when the researcher said “okay” instead of “skip training.”

## Experimental Design

**Fig. 2:**
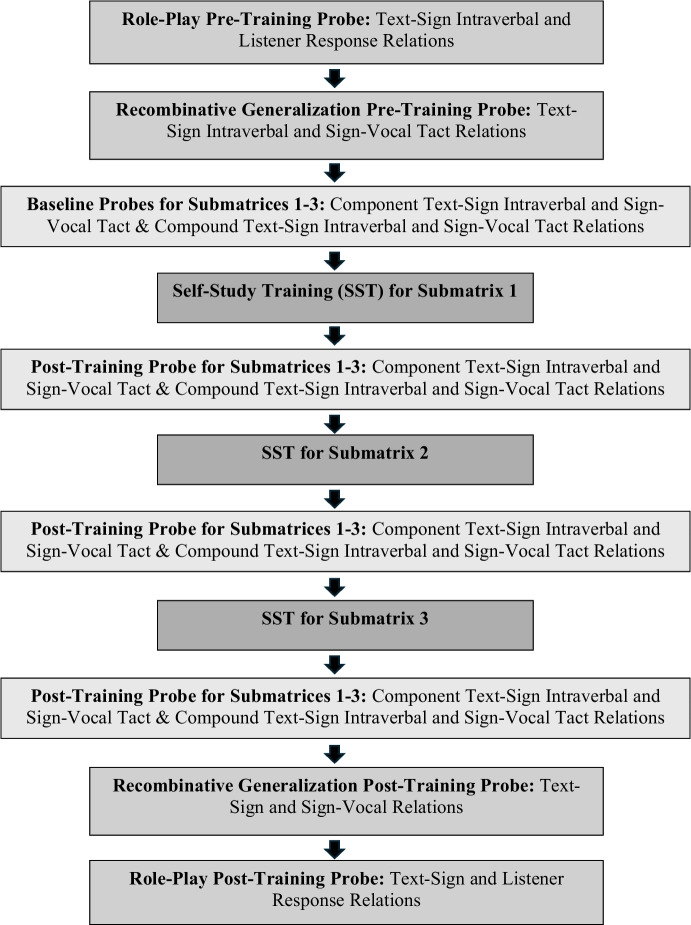
Flowchart of conditions

A multiple probe design across behaviors (i.e., submatrices) was applied to evaluate the effects of SST on the various dependent measures. Figure 2 displays a flowchart of the conditions in the study. In baseline, participants were tested on the text-sign intraverbals and sign-vocal tacts for the 12 component signs and the 12 compounds that compose the three submatrices. Following baseline, the participants completed SST with submatrix 1 diagonal targets. Once responding met SST mastery criteria, post-training probes were conducted for submatrices 1–3. These were considered post-training probes for the submatrix 1 and continued baseline probes for submatrices 2 and 3. Mastery criteria for the post-training probes were 100% correct responding on the text-sign intraverbals and sign-vocal tacts for both the compounds and components in submatrix 1. If these criteria were met, SST for submatrix 2 began. If these criteria were not met, the researcher repeated SST for submatrix 1. The participant experienced the same sequence of conditions (i.e., SST followed by post-training probes) for submatrices 2 and 3. If participant responding did not meet mastery criteria or responding did not maintain for a previously learned submatrix, the researcher repeated SST for all previously taught submatrices. For example, if participant responding did not meet mastery criteria during the post-training probe following submatrix 2 SST, the participant completed SST with the diagonal targets from both submatrices 1 and 2. This step was intended to promote stronger discrimination among trained compound signs (Grow et al., 2011; 2014). The probes for the recombinative generalization compounds and role plays were conducted before baseline and after the mastery criteria were met with submatrices 1–3.

## Role-Play Pre-training Probe

The 24 recombinative generalization compound targets (i.e., those not included within a submatrix) were divided into two groups of 12. The targets judged by easiest to respond to as a listener were assigned to the listener role play condition and the other 12 were assigned to the text-sign intraverbal role play condition. Each compound target was presented once in randomized order within a block of 12 trials. The researcher provided instructions to the participants on the importance of teaching and responding as a listener to signs when working with someone who communicates using sign.

This is a roleplay condition. When working with individuals who use sign language to communicate you will be tasked to use sign in a variety of ways. First, you will likely need to teach new signs to help expand vocabulary. Additionally, you will need to be able to respond to signed requests for items, activities, etc. If you are unable to provide an individual with what they are asking for, they might engage in challenging behavior or stop producing signs because it is not helping them get what they want. To replicate these real-life experiences, you will complete two different tasks. With the first, you will need to teach me the signs written on the provided notecards. If you are unsure on how to teach me the signs say, “I don’t know,” Once you have completed the notecards, I will take over signing. I will make a compound sign and you will respond. For example, if I were to sign “two crackers” you would want to grab two crackers. If you are unsure how to respond to the signs, say “I don’t know.” The experimenter will let you know when your responding meets criteria to move on.

During the role play the participant was seated across from the experimenter, without the computer present. To evaluate the text-sign intraverbal compound responses, the researcher gave the participant 12 notecards with different recombinative generalization compounds on each and said, “Teach me these by demonstrating the signs.” After participants attempted to teach the compound on each notecard or said, “I don’t know” they could flip to the next card, initiating the next trial. Following a sign attempt by the participant, the researcher provided neutral feedback by saying “okay.” The researcher did not imitate or otherwise respond to the demonstrated sign. Next, the researcher conducted the listener responses role play with the other 12 compound targets. The researcher placed all the items corresponding to the targets on the table in a messy array and gave the instruction, “Respond to my signs by doing what I ask.” During the role play the researcher emitted the compound signs and allowed the participant an opportunity to respond as a listener by providing the characteristic consequence. For example, when the researcher signed “where cookie” the participant was expected to point or otherwise indicate the location of the cookie within the array. The researcher provided a neutral acknowledgement following all responses (e.g., “ok”).

## Recombinative Generalization Pre-training Probe

The same 24 recombinative generalization compound targets (i.e., those not assigned to a submatrix) were evaluated as text-sign intraverbals and sign-vocal tacts. Targets were presented once in pre-randomized order in a block of 24 trials. The recombinative generalization probes began with general instructions regarding testing conditions.

This is a testing condition. There are two possible instructions you will encounter in this testing condition. The first, will include the words “sign this” followed by one or two words. When you see the printed word(s), try your best to produce the signs for those word(s). If you are unsure say “I don’t know.” The other type of instruction will include a video of single or compound signs along with the instruction to “label the sign.” Please attempt to label the sign out loud. If you are unsure say “I don’t know.” No feedback will be provided for correct or incorrect responses. The experimenter will let you know when your performance meets our criteria to move on.

The researcher presented 24 text-sign PowerPoint slides to the participant, one at a time. Each slide had the words “sign [modifier noun].” Next, 24 sign-vocal tact slides were presented one at a time. Each slide had printed instructions to “label the sign” with the corresponding video playing on a loop. Throughout the probe, the participant had access to the computer keys to move between slides at their desired pace; there was no maximum or minimum time for responding. As indicated in the instructions, the researcher did not provide differential feedback but indicated when the participant could progress to the next task.

## Baseline Probes for Submatrices 1–3

Baseline probes were conducted to evaluate performance on text-sign intraverbals and sign-vocal tacts for components and compounds within the submatrices prior to SST. The researcher began by informing the participant that this was another testing condition and asked if they wanted to hear the instructions again. If the participant indicated that they did not want to hear the instructions again, the researcher presented the designated PowerPoint slides in the order of conditions described in Fig. 2.

## Component Targets

The component targets on the axes of the matrix were evaluated in isolation. Each submatrix corresponded to two modifiers and two nouns (e.g., submatrix 1 included more, please, candy, and cookie) for a total of 12 component targets which were presented once in each trial block. The targets were evaluated as text-sign intraverbals with PowerPoint slides that included the typed direction to “sign [modifier/noun].” Next, the same targets were evaluated as sign-vocal tacts with slides that include the typed direction to “label the sign” with a video of the researcher demonstrating one of the components on a loop.

## Compound Targets

The 12 compound targets within submatrices 1–3 were evaluated as text-sign intraverbals and sign-vocal tacts following the procedures described above. Each submatrix included two diagonal targets (e.g., “more candy” and “please cookie”) and two non-diagonal targets (e.g., “more cookie” and “please candy”). Each target was presented once within a trial block and blocks were repeated until steady state responding was observed.

## Self-Study Training for Submatrices 1–3

During SST, the two diagonal compound targets within each submatrix were trained. Thus, for submatrix 1, only the signs for “more candy” and “please cookie” were presented. The researcher provided the following instructions detailing the condition.

This is a training condition. You will be presented with a compound sign. Try your best to produce the sign. If you sign it correctly, the instructor will inform you to press the green skip training button. Do not press this button unless instructed to do so. If any part of your sign is incorrect, the instructor will inform you to press the orange training button. After hitting the training button, you will be presented with a video of the sign repeated on a loop. Practice the sign as many times as you feel necessary. Make sure to attend to specific details of the sign including the handshape, the number of movements, the location of the sign, etc. If the sign doesn’t perfectly match the video, it will be marked as incorrect. After practicing, click the orange continue button. You will then have another opportunity to produce the sign independently. You will continue this process until you have signed the different component signs correctly and independently 3 times in a row.

The researcher started the session with an independent response opportunity for the text-sign intraverbal. The researcher presented a PowerPoint slide with written directions to sign the first of the diagonal compound targets (e.g., “sign [modifier noun],” see Supplemental Materials C Slide 1). This was considered an independent response opportunity. If the participant emitted a correct response, the researcher told them to press the “skip training” button which produced the independent opportunity for the text-sign intraverbal for the second diagonal compound target from the same submatrix. On either independent response opportunity, if the participant emitted an error, the researcher told them to press the “go to training” button which led to training slides for that compound sign (see Supplemental Materials C Slide 2). On the training slides, the printed words and a video of the researcher signing the compound sign on a loop appeared on the screen as in Longo et al. (2022). The participant could observe this slide for as long as they chose. When ready, the participants pressed the “continue” button. After the participant advanced from the training slide, they progressed to a slide that included only the printed words and instructions to sign (e.g., “sign [modifier noun],” see Supplemental Materials C Slide 3).

Data were only collected on the first independent response opportunities for the compounds, resulting in a total of two trials per block. If the participant emitted errors on these independent response opportunities for three consecutive blocks, the researcher repeated the instructions for the condition. SST for each submatrix was mastered when the participant independently signed both diagonal compounds correctly on the first independent response opportunity across three consecutive trial blocks. The duration of SST for each submatrix was measured from the presentation of the first independent response opportunity until mastery criteria were met.

## Post-training Probes for Submatrices 1–3

All procedures were identical to baseline. For each submatrix, mastery criteria consisted of 100% correct responding for the component (e.g., more, please, candy, cookie), diagonal compound (e.g., more candy, please cookie), and non-diagonal compound (e.g., more cookie, please candy) targets. If the mastery criteria were not met, SST was repeated. For submatrices 2 and 3, we also required 100% maintenance of prior submatrix responses. If correct responding did not maintain for a previously targeted submatrix, those diagonal targets would be included in the repeated SST condition (see Fig. 2).

## Recombinative Generalization Post-training Probe

All procedures were identical to the Recombinative Generalization Pre-training Probes. The performance criterion was 90% correct for both text-sign intraverbals and sign-vocal tacts. Performance below criterion resulted in repeating SST for all submatrices.

## Role-Play Post-training Probe

All procedures were identical to the Role-Play Pre-training Probes. The performance criterion was 90% for both text-sign intraverbals and listener responses. No contingencies were in place for performance below the criterion.

## Debrief and Social Validity

When all experimental conditions were complete, the researcher debriefed the participant on the purpose of the study. Additionally, the researcher informed the participants of the duration of the training and how many total signs they learned after combining all the components and the trained and untrained compounds. The participant was then asked to fill out a social validity questionnaire adapted from Longo et al. (2022). Table 2 includes the social validity questions. Participants rated how much they agreed with each statement using a Likert scale of 1–4 (disagree, somewhat disagree, somewhat agree, and agree). After completing the social validity questionnaire, the participants had as much time as they would like to ask any other questions or discuss their participation in the study.

**Table 2:** Social validity rankings for P1–P3

Statement:	Mean rating
This training helped me learn about forming signs and sign language.	4.00
I liked this self-study training method of learning.	3.33 (3–4)
The self-study training (when I watched the videos and practiced signing) took an appropriate amount of time to complete.	4.00
I would recommend this training procedure to other students.	3.67 (3–4)
The number of signs I learned during training helped me feel confident during our role-plays after training.	4.00

Rankings: 1= disagree, 2 = somewhat disagree, 3 = somewhat agree, and 4 = agree

## Results

**Fig. 3:**
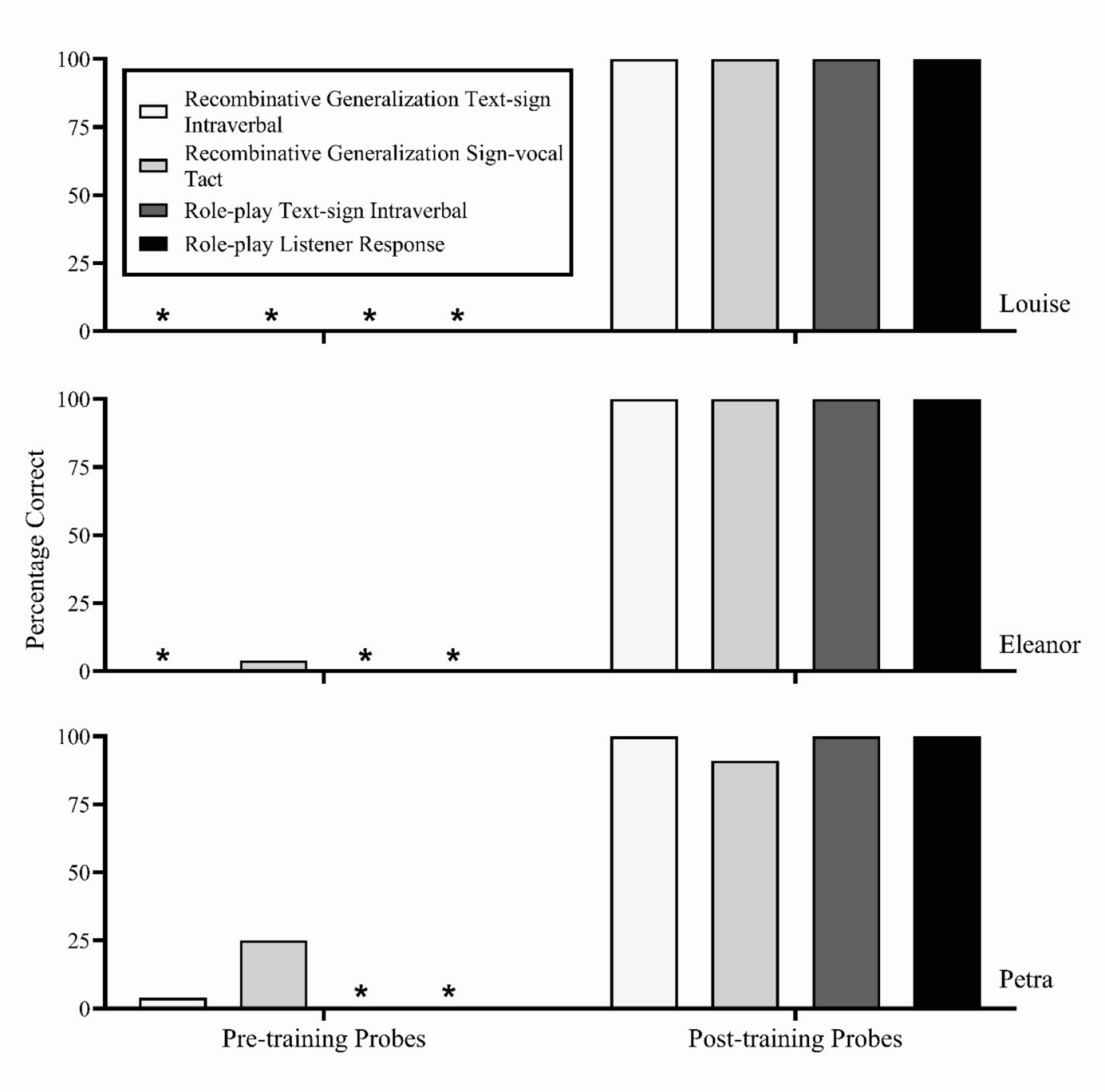
Performance on pre- and post-training probes across participants. *Note.* Asterisks denote 0% correct responding

**Fig. 4:**
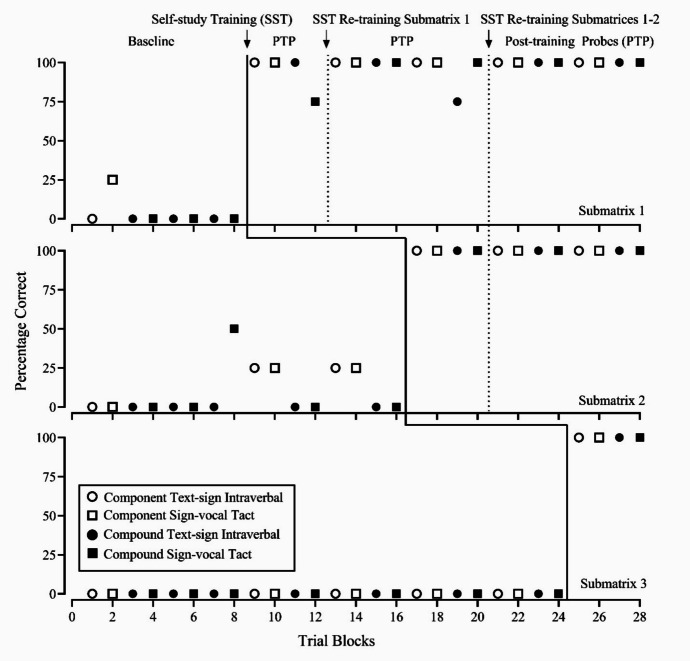
Performance on baseline and post-training probes for submatrices 1–3 for Louise

**Fig. 5:**
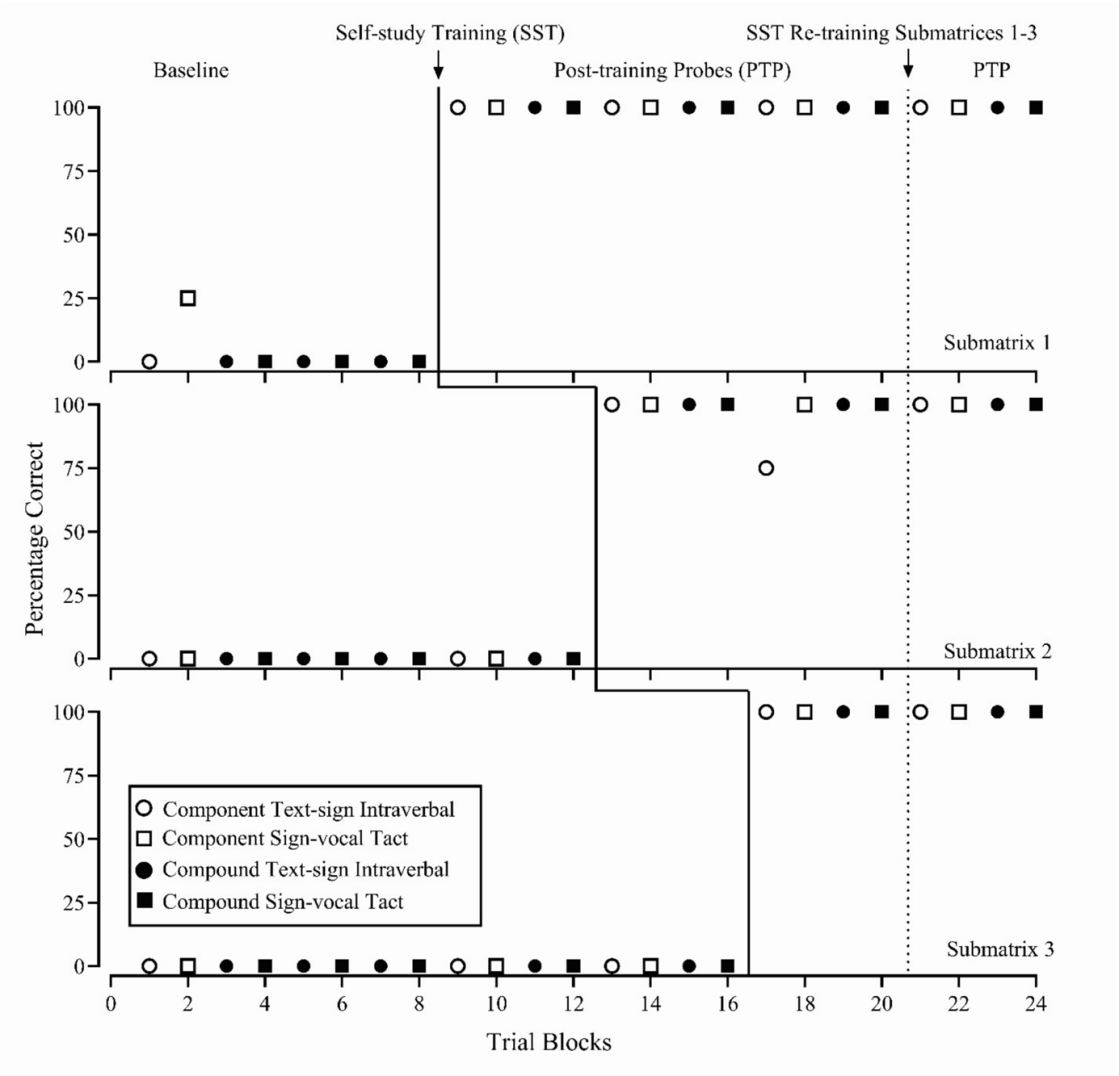
Performance on baseline and post-training probes for submatrices 1–3 for Eleanor

**Fig. 6:**
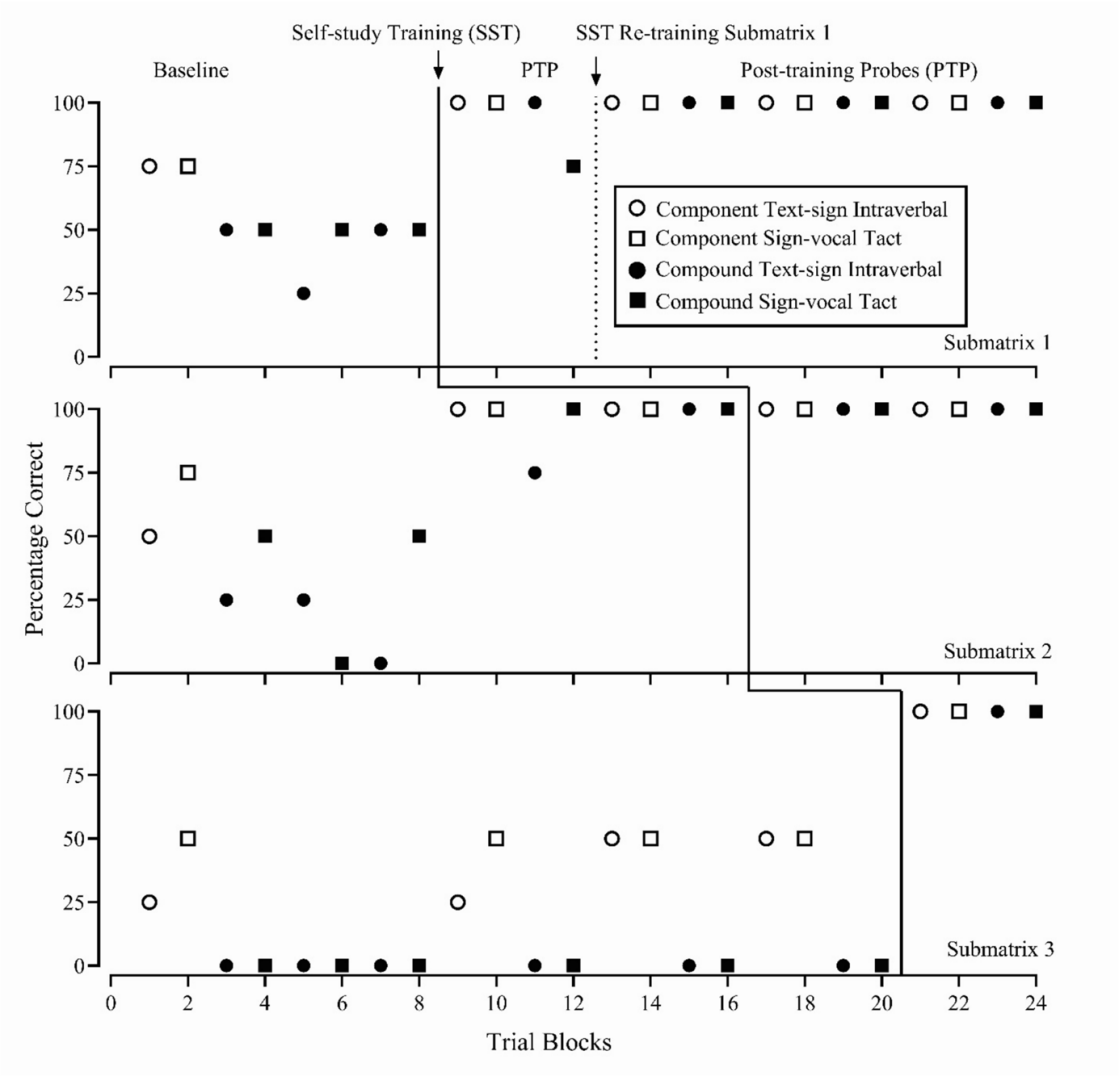
Performance on baseline and post-training probes for submatrices 1–3 for Petra

Results are organized by participant and in order of the conditions. Figure 3 includes performance during the role play conditions and recombinative generalization probes for all participants. Figures 4, 5 and 6 display baseline and post-training probes for submatrices 1–3 for each individual participant. Table 3 summarizes the results of SST.

**Table 3:** Participant training duration

Participant	Training phase	Duration (in min)	Number of trial blocks
Louise	Submatrix 1	4:03	5
	Submatrix 1 retraining	2:01	4
	Submatrix 2	6:52	7
	Submatrix 1 & 2 retraining	1:29	3
	Submatrix 3	4:11	4
	Total	18:36	23
Eleanor	Submatrix 1	2:30	5
	Submatrix 2	1:35	5
	Submatrix 3	1:47	5
	Submatrix 1, 2, & 3 retraining	1:53	4
	Total	7:45	19
Petra	Submatrix 1	3:44	9
	Submatrix 1 retraining	37 seconds	3
	Submatrix 2	1:05	3
	Submatrix 3	7:18	11
	Total	12:44	26

## Participant 1: Louise

Prior to SST, Louise emitted low levels of correct responding across trial types and conditions. Louise completed SST with submatrix 1 in five trial blocks but her responses on compound sign-vocal tacts trials during post-training probes were below the mastery criterion (75% in block 12), triggering a repetition of SST. Louise required four additional SST trial blocks before mastery criteria were met. During the SST sessions, Louise committed errors related to the movement and location of their nondominant hand during one-handed signs (i.e., holding nondominant hand near chest instead of down). When post-training probes were repeated (blocks 13–16), Louise demonstrated 100% correct responding on all trial types.

Louise completed SST with submatrix 2 in seven trial blocks. The second, third, and fourth SST trial blocks included errors with the nondominant hand. After three consecutive sessions with 50% responding, the researcher repeated the training condition instructions. Three trial blocks later, Louise’s responding met the SST mastery criteria. In the following post-training probes (blocks 17–20), Louise emitted 100% correct responses on all submatrix 2 targets but responding for submatrix 1 compound text-sign intraverbal fell below the mastery criterion, with a score of 75%. SST was repeated with both submatrix 1 and 2 targets. Each trial block in re-training consisted of a presentation of all four previously learned compounds (i.e., more candy, please cookie, want glue, and help doll). Re-training required four SST trial blocks but her responding met the mastery criterion in the following post-training probes (blocks 21–24).

Louise completed SST with submatrix 3 in four trial blocks, and 100% correct responding was observed with all submatrices during the post-training probes (blocks 25–28). During the recombinative generalization and role-play probes, Louise emitted 100% correct responses across trial types. The total duration of SST for Louise was 18:36 minutes.

## Participant 2: Eleanor

Eleanor also emitted low levels of correct responding across trial types and probe conditions prior to SST. Eleanor completed SST with submatrix 1 after five trial blocks and demonstrated mastery level responding for all trial types in the subsequent post-training probes. Eleanor completed SST with submatrix 2 after five trial blocks and demonstrated 100% correct responding across all trial types for submatrices 1 and 2 during post-training probes. Eleanor completed SST with submatrix 3 after five trial blocks. During post-training probes (blocks 17–20), correct responding occurred on all trials for submatrix 1 and 3 but fell below criterion for submatrix 2 text-sign intraverbal targets. SST was repeated with submatrices 1, 2, and 3 targets, lasting four trial blocks. Each trial block in re-training consisted of a presentation of all six previously learned compounds (i.e., more candy, please cookie, want glue, help doll, no paper, and where train). When the post-training probes were repeated (blocks 21–24), 100% correct responding was observed for all submatrices across trial types. During the recombinative generalization and role-play probes, Eleanor emitted 100% correct responding across trial types. The total duration of SST for Eleanor was 7:45 minutes.

## Participant 3: Petra

Petra emitted low levels of correct responding during the role-play and recombinative generalization probes. However, in the submatrices 1–3 baselines, highly variable performance was observed across trial types as Petra attempted responses on nearly all trials. As responding was below mastery criteria, intervention proceeded. Petra completed SST with submatrix 1 in nine trial blocks, but in the post-training probes her compound sign-vocal tact responding did not meet mastery criteria, with a score of 75%. But Petra engaged in mastery or near mastery level performance with submatrix 2 targets. Petra’s errors with submatrices 1 and 2 targets in baseline were due to discrimination errors between “candy” and “doll.” SST was repeated for submatrix 1, lasting three trial blocks. Petra did not emit any errors during re-training. During the subsequent post-training probes (blocks 13–16), Petra emitted 100% correct responses on both submatrix 1 and submatrix 2 targets.

Although mastery criteria were already achieved for submatrix 2 targets in baseline, the researcher conducted SST for submatrix 2 to strengthen responding through differential reinforcement. During SST, Petra emitted correct responses on each first attempt, concluding training in three trial blocks and mastery level responding was maintained across submatrices 1 and 2 in the following post-training probes (blocks 17–20).

Petra completed SST with submatrix 3 in 11 trial blocks. After three consecutive sessions with consistent errors on the “where train” target, the researcher repeated the training condition instructions. Following the repeated instructions, Petra’s responding improved and mastery criteria were met after four more trial blocks. During SST, she consistently practiced the sign “train” even though she was erroring on the sign “where.” During the final post-training probes (blocks 21–24), Petra emitted 100% correct responses for all submatrices and trial types. Criterion-level responding was observed during the recombinative generalization and role-play probes. The total duration of SST for Petra was 12:44 minutes.

## Social Validity

Table 2 displays results of the social validity survey. Participants agreed with the statements “This training helped me learn about forming signs and sign language” (*M* = 4.00), “The self-study training (when I watched the videos and practiced signing) took an appropriate amount of time to complete” (*M* = 4.00), “I would recommend this training procedure to other students” (*M* = 3.67, range 3–4), and “The number of signs I learned during training helped me feel confident during our role-plays after training” (*M* = 4.00). Participants somewhat agreed with the statement “I liked this self-study training method of learning” (*M* = 3.33, range 3–4).

## Discussion

Results from the current study replicate findings from Longo et al. (2022) by demonstrating the efficacy of SST for training signs and establishing untrained signs across response modalities and contexts. By the completion of the study, participants emitted all 36 compound and 12 component signs under control of textual stimuli (i.e., text sign-intraverbal) and tacted at least 34 of the 36 compound signs and all 12 of the component signs (i.e., sign-vocal tact). In the role plays, participants emitted 12 compound signs (i.e., text-sign intraverbal) and responded as listeners to 12 compound signs. These results extend Longo et al. through expansion of the matrix and inclusion of a role play context.

The mean duration of SST was 13:01 minutes to learn the six diagonal compound signs, approximately two minutes per compound or one minute per component. This closely mirrors findings in Longo et al. (2022) in which the mean duration of SST was eight minutes to learn eight component signs. Notably, the mean duration of SST in the present study was still shorter than the mean duration reported for the equivalence-based instruction group in the Longo et al. study (22.5 minutes). Collectively, these results join Longo et al. to suggest that self-study can be an efficient method for learning compound or component signs.

The efficiency and efficacy of the SST used in the Longo et al. (2022) and the present study may be attributed to strategic instructional design that leveraged the adult participants’ behavioral cusps. Longo et al. posited that SST may have functioned as a type of respondent-type training procedure (Leader et al., 1996), in which stimuli become discriminative for one another through temporal contiguity (for review see Brown et al., 2023). This differs from equivalence-based instructional approaches that train conditional discriminations through matching-to-sample and differential reinforcement. However, this analysis neglects the role of active participant responding that may be occurring during SST. During SST, participants may have been reacting to the text vocally as speakers (i.e., saying “more cookie”) and emitting duplic responding by imitating the signs in the presence of the text (anecdotally, observed with Petra). As they emitted speaker behavior in the presence of the signs, through transfer of stimulus control the signs could evoke the vocal speaker responses (Horne & Lowe, 1996; Miguel, 2016; 2018), accounting for the sign-vocal tacts. Additionally, as participants could behave discriminatively as listeners to themselves as speakers (Horne & Lowe, 1996; Miguel, 2016; 2018), signs could function to occasion listener responding, as in the role plays. Importantly, the goal of this study was not to evaluate these mechanisms but to use them to quickly produce many behaviors. Future studies could consider requiring participants to talk aloud during SST (e.g., Wulfert et al., 1991) or use gaze-tracking software (e.g., Braaten & Arntzen, 2023) to more closely evaluate participant responding during SST.

This study extended the application of a matrix training approach used by Longo et al. (2022) with the use of submatrices and non-overlap training. Axe and Sainato (2010) included submatrices within a larger matrix that progressively expanded by one or two diagonal targets and incorporated recombinations with previously trained components. This tactic resulted in varying numbers of targets per submatrix, and thereby non-equated sets of behaviors across tiers. In the present study, only the two diagonal and two non-diagonal targets were included in each of the three submatrices so that each had the same number of targets (four). Only these 12 targets were probed throughout training, saving time while providing evidence of recombinative generalization with performance on the non-diagonal targets. The remaining 24 targets, which required recombination of responses across submatrices, were only evaluated once strong, discriminated responding was already established on a smaller scale. Overall, this tactic may prove useful when including larger matrices under resource-sensitive conditions.

The use of non-overlap training with compounds did not prove to be more efficient in terms of time spent in SST; however, it did involve fewer teaching materials. Longo et al. (2022) used SST to teach eight component skills, requiring the preparation of eight training slides. In a six-by-six matrix, this would mean 12 slides. With the use of overlap training, only six slides were needed. Though this difference is negligible for a research study, in practice this may prove to be an important time-savings. Of note, practitioners could develop SST materials using only slides and videos from the internet, aligned with the sign repertoires of each of their clients. As new signs are mastered, the matrix used to organize the targets could simply grow through the addition of new SST slides. Future studies could test this contention by measuring the time to create the materials and incorporating that into the overall evaluation of efficiency.

Kemmerer et al. (2021) reported that across the growing matrix literature, adult participants were included in only 18% of the studies and none were neurotypical (note, diagnosis was not reported in all studies). Inclusion of neurotypical adult participants, expected to produce recombinative generalization, could advance matrix research through establishing boundary conditions and optimal practices. Content relevant to staff member training could continue to be a viable area for further investigations.

There are several limitations impacting these findings. First, Petra’s results indicate that the acquisition of text-sign intraverbals and sign-vocal tacts may occur from mere exposure and repetition of both relations, a testing threat to internal validity (Ledford, 2017). Meeting mastery criteria before SST suggests performance across submatrices was not independent (Ledford & Zimmerman, 2023). This prevents a strong demonstration of control as we did not verify that performance with submatrix 2 would remain unchanged until SST began. Functional control was demonstrated more clearly with submatrix 3, as no compound responses were observed until SST was introduced. Future studies could consider omitting the sign-vocal tact topography from the ongoing probes to prevent extended exposures to the signs during baseline.

Conclusions regarding the generality of these findings are limited by inclusion of only three participants and choices related to participant characteristics and settings. Specifically, none of the participants in the study were employed as staff members teaching sign to individuals using AAC and the roleplay was conducted with a vocal-verbal communicator on a university campus. These choices were made for convenience to the researchers at the cost of ecological validity. Of note, the participant characteristics did overlap with common racial, gender, and age-related demographics of individuals that hold a credential as a Registered Behavior Technician © (Behavior Analyst Certification Board, (Behavior n.d). Future studies should prioritize recruiting staff members working in applied settings with patients that use sign as AAC to increase external and ecological validity. Evaluations with larger batches of participants would speak to the scalability of the approach within the workplace (e.g., Parsons et al., 2017).

Sign as a form of AAC has been recommended given the inclusion of distinct topographical responses (Michael, 1985; Sundberg & Michael, 2001; Sundberg & Partington, 1998), no need for equipment (Bonvillian et al., 2020), and potential implementation in infancy (Howlin, 2003). Despite the advantages of sign, the necessity for implementers to learn sign may serve as a barrier to its use (Bonvillian et al., 2020; Brodhead et al., 2020; Sundberg & Sundberg, 1990). Results from Longo et al. (2022) and the present study address this barrier by showing that adults can learn to behave as speakers and listeners with signs after very brief training when effective instructional design tactics are used. However, this approach does not address barriers related to the recognizability of signs with untrained community members (Broadhead et al., 2020). Thus, the contexts in which the user spends their time and the likelihood of ongoing support from trained staff must continue to play a critical role in the selection of the AAC modality (Broadhead et al., 2020; Beukelman & Light, 2020).

There is still more to learn to determine the most effective and efficient methods for staff training in AAC modalities. As stated by Chadwick and Jolliffe (2009), “it is likely that, if the staff are not adequately trained to recognize the person’s own signs, they may disregard the communicative actions of individuals who use AAC” (p. 35). It is our responsibility to ensure that the individuals we serve can continuously communicate their wants and needs to a verbal community that has been conditioned to reinforce their responses (Skinner, 1957).

## Supplementary Information

Below is the link to the electronic supplementary material.

Supplementary file1 (DOCX 3221 KB)

## Data Availability

The data that support the findings of this study are available from the corresponding author upon reasonable request.
