# Supplementary material for: The Use of Self-Study and Matrix Training to Teach Sign Language
Source: Anal Verbal Behav. 2026 Apr 27;42(1):84–108. doi: 10.1007/s40616-026-00228-2 (PMC13469049; doi:10.1007/s40616-026-00228-2)
Supplement: Supplementary file 1 [file 40616_2026_228_MOESM1_ESM.docx]

**Supplemental Materials A**

*Recombinative Generalization Probe Slides*

*1. Text-Sign Intraverbal	2. Sign-Vocal Tact*

*This format will remain the same for all 24 Recombinative Generalization Slides

**Supplemental Materials B**

*Submatrices 1-3 Baseline and Post-Training Probe Slides*

*1. Component Text-Sign Intraverbal	2. Compound Text-Sign Intraverbal*

*3. Component and Compound Sign-Vocal Tact*

*This format will remain the same for all Baseline and Post-Training Probe Slides

**Supplemental Materials C**

*Self-Study Training Slides*

*1. Independent Sign Opportunity	2. Self-Study Training*

*3. Independent Sign Opportunity*

*This format will remain the same for all Self-Study Teaching Slides

**Supplemental Materials D**

*Pre- and Post-training Probe Procedural Fidelity Data Sheet*

| Procedural Fidelity | | | |
| --- | --- | --- | --- |
| **Condition: Role-Play** | | Session #: | Researcher: |
| Participant: | | Data Collector: | Score: |
| 1 | Instructions Provided (If applicable) | |  |
| 2 | Signs the correct targets for Listener Response Probes | |  |
| 3 | Provides no feedback for responses | |  |
| 4 | Only interacts as appropriate to protocol | |  |
| NA= Not Applicable, + = Correct, - = Incorrect | | | |
|  | | | |
| **Condition: Recombinative Generalization** | | Session #: | Researcher: |
| Participant: | | Data Collector: | Score: |
| 1 | Instructions Provided (If applicable) | |  |
| 2 | Provides no feedback for responses | |  |
| 3 | Only interacts as appropriate to protocol | |  |
| NA= Not Applicable, + = Correct, - = Incorrect | | | |
|  | | | |

**Supplemental Materials E**

*Submatrices 1-3 Procedural Fidelity Data Sheet*

| Submatrices 1-3 Procedural Fidelity | | | |
| --- | --- | --- | --- |
| Condition: | | Session #: | Researcher: |
| Participant: | | Data Collector: | Score: |
| 1 | Instructions provided (If applicable) | |  |
| 2 | Provides no feedback for responses | |  |
| 3 | Only interacts as appropriate to protocol | |  |
| NA= Not Applicable, + = Correct, - = Incorrect | | | |
|  | | | |

**Supplemental Materials F**

*Self-study Training Procedural Fidelity Data Sheet*

| Procedural Fidelity | | | |
| --- | --- | --- | --- |
| Condition: | | Session #: | Researcher: |
| Participant: | | Data Collector: | Score: |
| 1 | Instructions Provided (If applicable) | |  |
| 2 | Follows protocol when deciding if participant needs SST | |  |
| 3 | Provides no feedback for responses | |  |
| 4 | Only interacts as appropriate to protocol | |  |
| NA= Not Applicable, + = Correct, - = Incorrect | | | |
|  | | | |
